# Combination of betulinic acid and chidamide synergistically inhibits Epstein-Barr virus replication through over-generation of reactive oxygen species

**DOI:** 10.18632/oncotarget.18661

**Published:** 2017-06-27

**Authors:** Haibing Yu, Hongyu Zhang, Zhigang Chu, Qiongfang Ruan, Xueru Chen, Danli Kong, Xiaodong Huang, Huawen Li, Huanwen Tang, Hongjin Wu, Yifei Wang, Weiguo Xie, Yuanling Ding, Paul Yao

**Affiliations:** ^1^ School of Public Health, Guangdong Medical University, Dongguan 523808, PR China; ^2^ Department of Hematology, Peking University Shenzhen Hospital, Shenzhen 518036, PR China; ^3^ Institute of Burns, Tongren Hospital of Wuhan University, Wuhan 430060, PR China; ^4^ Beijing Haidian Hospital, Haidian Section of Peking University 3^rd^ Hospital, Beijing 100080, PR China; ^5^ Guangzhou Biomedical Research and Development Center, Jinan University, Guangzhou 510632, PR China

**Keywords:** betulinic acid, chidamide, Epstein-Barr virus, reactive oxygen species, histone deacetylase inhibitor

## Abstract

Epstein-Barr virus (EBV) has widely infected more than 90% of human populations. Currently, there is no efficient way to remove the virus because the EBV carriers are usually in a latent stage that allows them to escape the immune system and common antiviral drugs. In the effort to develop an efficient strategy for the removal of the EBV virus, we have shown that betulinic acid (BA) slightly suppresses EBV replication through SOD2 suppression with subsequent reactive oxygen species (ROS) generation and DNA damage in EBV-transformed LCL (lymphoblastoid cell line) cells. Chidamide (CDM, CS055), a novel histone deacetylase inhibitor (HDACi), could significantly switch EBV from the latent stage to the lytic stage with increased gene expression of BZLF1 and BMRF1, but has a small effect on EBV replication due to the suppression effect of CDM-mediated ROS generation. Interestingly, a combination of BA and CDM synergistically inhibits EBV replication with ROS over-generation and subsequent DNA damage and apoptosis. Overexpression of SOD2 diminishes this effect, while SOD2 knockdown mimics this effect. An *in vivo* xenograft tumor development study with the tail vein injection of EBV-transformed LCL cells in nude mice proves that the combination of BA and CDM synergistically increases superoxide anion release in tumor tissues and suppresses EBV replication and tumor growth, and significantly prolongs mouse survival. We conclude that the combination of BA and CDM could be an efficient strategy for clinical EBV removal.

## INTRODUCTION

Epstein-Barr virus (EBV) is a human γ-herpesvirus that infects more than 90% of human populations. Since most EBV carriers have a latent infection, they do not display any obvious clinical symptoms because EBV is normally well controlled by the immune system [1]. EBV is etiologically associated with a number of human malignancies, including Burkitt’s lymphoma, Hodgkin’s lymphoma, non-Hodgkin lymphoma, nasopharyngeal carcinoma (NPC), posttransplant lymphoproliferative disorder (PTLD), and some sporadic cancers of the gastrointestinal tract and breast [1, 2]. Current anti-herpes virus drugs, including nucleoside analogs ganciclovir (GCV) and acyclovir, are usually inefficient in removing the EBV virus from patients, as EBV maintains a latent stage and lytic-phase proteins are required to convert these pro-drugs to active antiviral drugs [3]. Development of an efficient anti-EBV removal strategy is still a challenge.

Betulinic acid (BA) is a natural product that is derived from plant sources, and has been characterized as a highly selective inhibitor of human melanoma cell [4] and tumor growth [5] through induction of apoptosis [6]. BA can inhibit the hepatitis B virus through SOD2 suppression with subsequent oxidative stress [7], indicating that BA may be a potential candidate for development of anti-EBV drugs. Some histone deacetylase inhibitors (HDACi) [8], such as MS275 [9], can be a potent activator in switching EBV from the latent to lytic stage, and subsequently sensitizes lymphoma cells to nucleoside antiviral agents [3]. Chidamide (CDM, CS055) is a novel benzamide-type HDACi, a synthetic analogue of MS-275, and is currently in clinical trials for leukemia in China [5, 10]. CDM (CS055) can induce significant cell-cycle arrest, resulting in the inhibition of cell proliferation and apoptosis in leukemia cells [11], while there is no report about the effect of CDM (CS055) on EBV.

Manganese superoxide dismutase (SOD2) is an antioxidant enzyme located in the mitochondria that could scavenge superoxide anions (O2^.-^) to hydrogen peroxide. Suppression of SOD2 expression may lead to the over-generation of reactive oxygen species (ROS) from the mitochondria, which subsequently triggers mitochondrial dysfunction and apoptosis [12, 13]. Altered SOD2 expression is considered both beneficial and detrimental. For instance, overexpression of SOD2 could be protective from ROS-mediated cell damage, but it may also increase the invasiveness of tumors and achieve higher possibility of infection (14-16), while SOD2 suppression may inhibit virus infection due to the over-generation of ROS [7, 17].

EBV infection is usually latent with few viral function proteins expressed, including latent membrane proteins (LMP), such as LMP1 and EBNA1 (Epstein–Barr nuclear antigen 1) [18]. LMP-1, which is encoded by the BNLF-1 gene, is considered one of the major oncoproteins among the EBV-expressed proteins [19]. EBNA1 is a viral encoded DNA binding protein that is essential for the stable maintenance of the EBV circular genome during latent infection. EBNA1 is consistently expressed in all EBV-associated tumors, and is thought to provide a survival function in addition to the maintenance of the viral genome [20, 21]. The EBV proteins EBNA1 and LMP1 are two key proteins for viral DNA replication and tumorigenesis, and suppression of these two proteins are an important step for the inhibition of EBV-associated tumor growth. Activation of the EBV lytic cycle is mediated through the combined actions of ZEBRA and Rta, the products of the viral BZLF1 and BRLF1 genes. During latency, these two genes are tightly repressed [22]. The EBV BMRF1 protein is a DNA polymerase processivity factor, and is essential for lytic replication of the EBV genome [23]. Expression of BZLF1 and BMRF1 is a marker of the activation of EBV from the latent stage, and the suppression of those two genes has potential clinical application for EBV DNA removal.

In the effort to develop an efficient anti-EBV removal strategy, we found that BA could slightly inhibit EBV replication in EBV-transformed LCL (lymphoblastoid cell line) cells through SOD2 suppression and subsequent oxidative stress [24]. Chidamide (CS055) could activate EBV from a latent to lytic stage with the addition of ROS generation, while it showed little effect in inhibiting EBV replication. On the other hand, combination of BA and CDM synergistically increased ROS generation with subsequent DNA damage and apoptosis, and significantly inhibited EBV replication. The *in vivo* xenograft tumor development study through tail vein injection of EBV-transformed LCL cells showed that a combination of BA/CDM synergistically increased the superoxide anion release in EBV-transformed LCL tumor tissues [25], and significantly suppressed EBV replication and tumor growth with prolonged mouse survival. Overexpression of SOD2 diminished this inhibition effect, while SOD2 knockdown mimicked this effect. We conclude that a combination of BA/CDM would be an efficient EBV removal strategy through overgeneration of ROS.

## RESULTS

### Betulinic acid (BA) suppresses SOD2 expression with increased ROS formation in LCL cells

**Figure 1:**
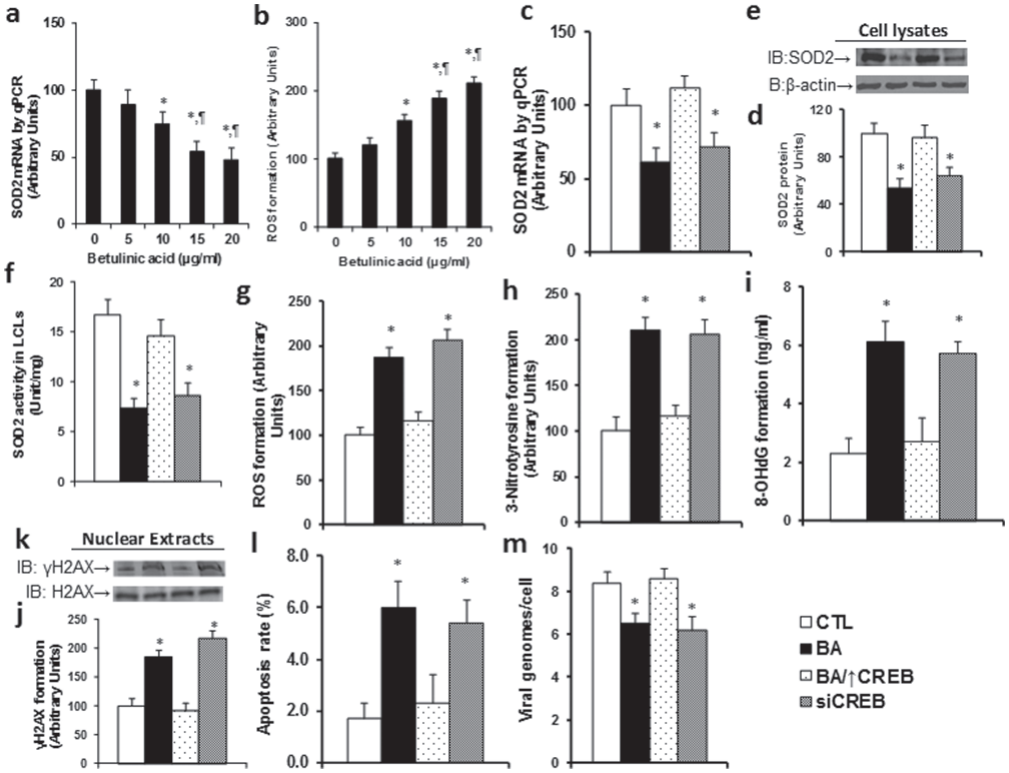
Betulinic acid (BA) suppresses SOD2 expression with increased ROS formation in LCL cells **(a, b)** The EBV-transformed LCL cells were treated with different concentrations of betulinic acid (BA) for 24 hours, and the cells were harvested for analysis. **(a)** The SOD2 mRNA by qPCR, n=4. **(b)** The ROS (reactive oxygen species) formation, n=4. *, *P*<0.05, vs 0μg/ml betulinic acid group; ¶, *P*<0.05, vs 10μg/ml betulinic acid group. **(c-m)** The LCL cells were treated by the control (CTL), BA (15μg/ml betulinic acid), 15μg/ml BA plus CREB overexpression (BA/↑CREB), or CREB knockdown (siCREB) for 24 hours, and then the cells were harvested for further analysis. **(c)** SOD2 mRNA by qPCR, n=4. **(d)** SOD2 protein quantitation, n=5. **(e)** Representative picture of Western Blots for **(d)**. **(f)** SOD2 activity assay, n=5. **(g)** ROS formation, n=5. **(h)** 3-Nitrotyrosine (3-NT) formation, n=4. **(i)** 8-OHdG formation, n=4. **(j)** γH2AX formation, n=5. **(k)** Representative picture of Western Blots for **(j)**. **(l)** Apoptosis rate by TUNEL assay, n=5. **(m)** EBV viral genomes/cell by qPCR, n=4. *, *P*<0.05, vs CTL group. Results are expressed as mean ± SEM.

We first measured the mRNA expression of SOD2 after betulinic acid treatment with different doses in EBV-transformed LCL cells. In Figure 1a, the SOD2 expression decreased by almost 50% in the presence of 15μg/ml BA. We then measured the levels of reactive oxygen species (ROS) in LCLs, and found that ROS formation increased following the increased BA doses (see Figure 1b). It has been reported that SOD2 suppression is due to BA-induced CREB dephosphorylation [7], and we wanted to see whether CREB affects SOD2 expression. Our results showed that BA-mediated SOD2 suppression, including mRNA level (see Figure 1c) and protein level (see Figure 1d, 1e), was completely normalized by CREB overexpression (BA/↑CREB), while CREB knockdown (siCREB) mimicked the effect. We then measured SOD2 activity, and effects similar to those of SOD2 protein were observed (see Figure 1f). We then measured oxidative stress, including ROS (see Figure 1g) and 3-nitrotyrosine formation (see Figure 1h), DNA damage, including 8-OHdG (see Figure 1i) and γH2AX formation (see Figure 1j, 1k), and apoptosis rate (see Figure 1l). It showed that BA significantly increased oxidative stress, DNA damage and apoptosis rate, CREB overexpression completely diminished the effect, and CREB knockdown mimicked the BA effect. We finally measured the EBV viral DNA copies (see Figure 1m), and it showed that BA slightly inhibited EBV replication, CREB overexpression diminished this effect, and CREB knockdown mimicked the effect. These results indicate that BA-mediated EBV inhibition is due to BA-induced SOD2 suppression and the subsequent oxidative stress.

### Chidamide (CDM) treatment activates EBV lytic gene expression through increased H3 acetylation in LCL cells

**Figure 2:**
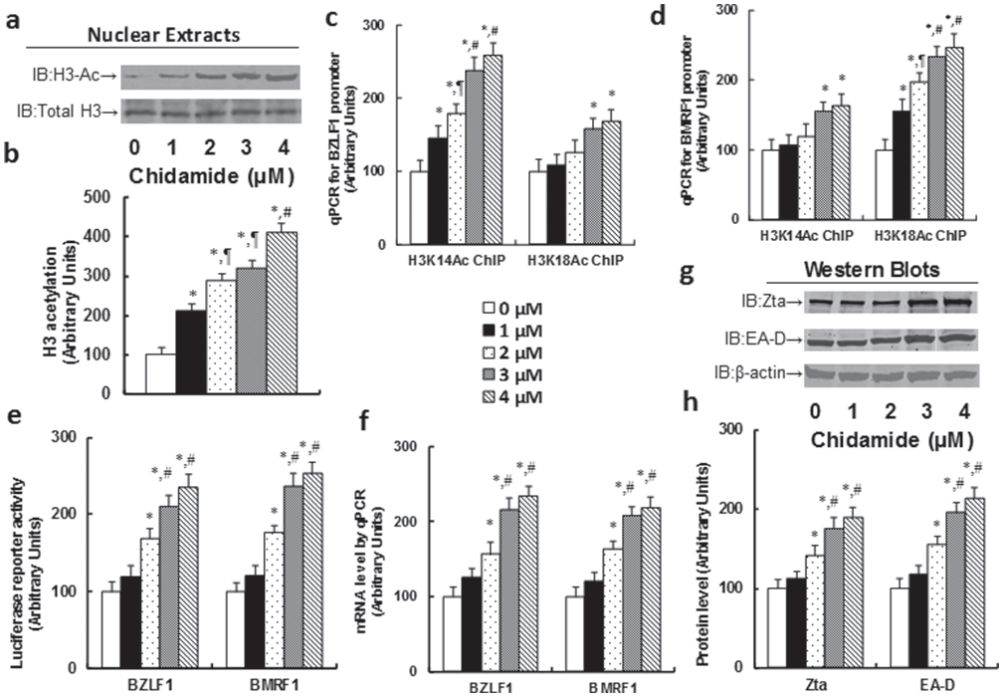
Chidamide (CDM) treatment activates the EBV lytic gene expression through increased H3 acetylation in LCL cells The LCL cells were treated with different concentrations of chidamide (CDM) for 24 hours, and the cells were harvested for analysis. **(a)** Representative pictures for H3 acetylation. **(b)** H3 acetylation quantitation from Western Blots, n=5. **(c)** ChIP analysis on BZLF1 promoter, n=4. **(d)** ChIP analysis on BMRF1 promoter, n=4. **(e)** Luciferase reporter assay for BZLF1 and BMRF1, n=5. **(f)** mRNA level by qPCR, n=4. **(g)** Representative picture of Western Blots. **(h)** Protein quantitation for Zta and EA-D, n=5. *, *P*<0.05, vs 0 μM Chidamide; ¶, *P*<0.05, vs 1 μM Chidamide; #, *P*<0.05, vs 2 μM Chidamide. Results are expressed as mean ± SEM.

We first measured the effect of chidamide (CDM) on histone acetylation with different CDM doses. In Figure 2a, 2b, H3 acetylation increased following the increase of CDM doses. We then measured the epigenetic changes of H3 acetylation on BZLF1 (see Figure 2c) and BMRF1 (see Figure 2d) promoters. It showed that the binding ability of H3 acetylation on K14 (H3K14Ac) increased significantly with CDM dose response on the BZLF1 promoter, while it had only a small effect on the BMRF1 promoter. On the other hand, the binding ability of H3 acetylation on K18 (H3K18Ac) had a small effect on the BZLF1 promoter, while effects increased significantly on the BMRF1 promoter. We then measured the luciferase reporter activity of BZLF1 and BMRF1 (see Figure 2e). It showed that both gene activities increased significantly with the CDM dose response. We finally measured the gene expression of BZLF1 and BMRF1, including mRNA level (see Figure 2f) and protein level (see 2g, 2h). The results showed that gene expression increased significantly with the CDM dose response.

### Chidamide (CDM) treatment potentiates ROS formation, DNA damage and apoptosis, and slightly increases EBV replication in LCL cells

**Figure 3:**
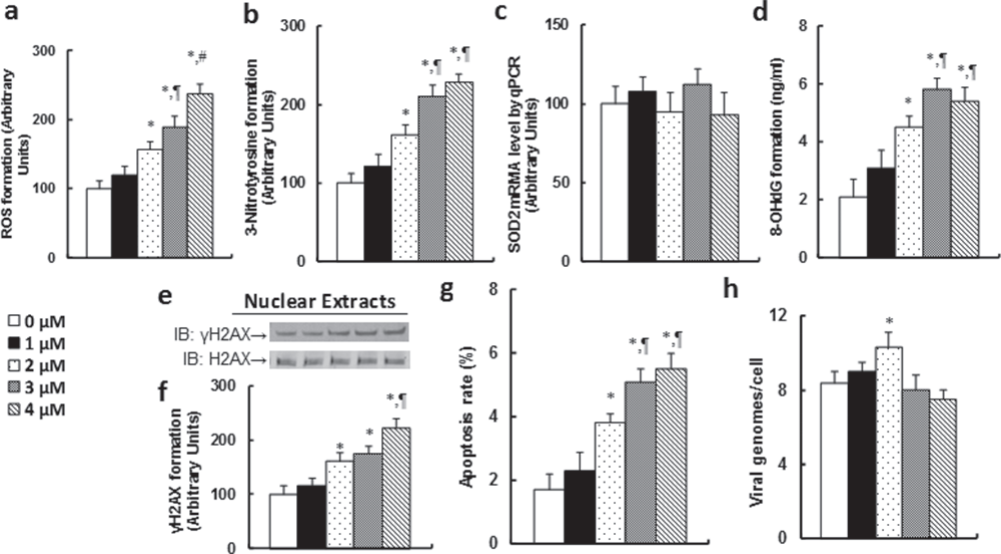
Chidamide (CDM) treatment potentiates ROS formation, DNA damage and apoptosis, and slightly increases EBV replication in LCL cells The LCL cells were treated with different concentrations of chidamide (CDM) for 24 hours, and the cells were harvested for analysis. **(a)** ROS formation, n=5. **(b)** 3-Nitrotyrosine formation, n=5. **(c)** SOD2 mRNA by qPCR, n=4. **(d)** 8-OHdG formation, n=5. **(e)** Representative pictures for γH2AX formation. **(f)** Protein quantitation for **(e)**, n=5. **(g)** Apoptosis rate by TUNEL assay, n=5. **(h)** EBV viral genomes/cell by qPCR, n=4. *, *P*<0.05, vs 0 μM Chidamide; ¶, *P*<0.05, vs 2 μM Chidamide; #, *P*<0.05, vs 3 μM Chidamide. Results are expressed as mean ± SEM.

We first measured the ROS (see Figure 3a) and 3-Nitrotyrosine (see Figure 3b) formation in LCL cells, and the results showed that both increased significantly with the CDM dose response. We then measured the SOD2 mRNA expression, and found that there was no change in expression with CDM treatment (see Figure 3c). We also measured the DNA damage and apoptosis that resulted from the CDM treatment, including the 8-OHdG (see Figure 3d), γH2AX formation (see Figure 3e, 3f) and apoptosis rate (see Figure 3g). Our results showed that DNA damage and apoptosis rate increased significantly with the CDM dose response. We then measured the EBV DNA copies. The results showed that EBV DNA copies increased slightly following CDM treatment, but when the CDM treatment reached 4μM, the EBV DNA copies did not increase but rather decreased significantly compared to the 3μM CDM treatment, indicating that high doses of CDM treatment-induced ROS formation may suppress EBV DNA replication.

### Combination of betulinic acid (BA) and chidamide (CDM) synergistically potentiates ROS formation, DNA damage, apoptosis and mitochondrial dysfunction, while SOD2 overexpression diminishes this effect

**Figure 4:**
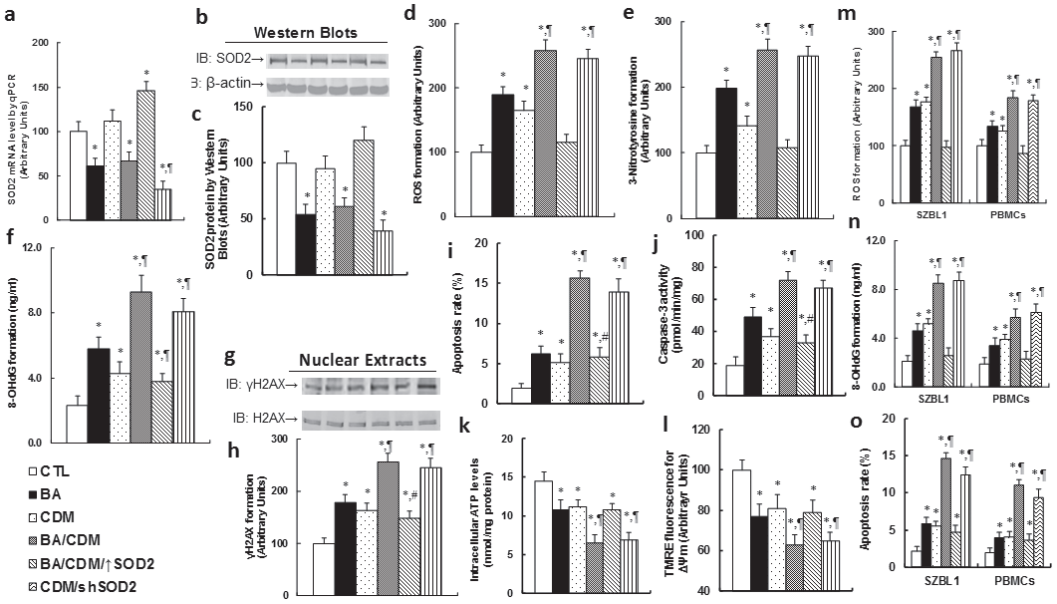
Combination of betulinic acid (BA) and chidamide (CDM) synergistically potentiates ROS formation, DNA damage and apoptosis, while SOD2 overexpression diminishes this effect **(a-l)** The EBV-transformed LCL cells were treated with the control (CTL) alone, 15μg/ml BA alone (BA), 3μM CDM alone (CDM), a combination of BA and CDM (BA/CDM), a combination of BA and CDM treatment in SOD2 overexpression cells (BA/CDM/↑SOD2), or CDM treatment in SOD2 knockdown cells (CDM/shSOD2) for 24 hours, and the cells were harvested for further analysis. **(a)** SOD2 mRNA level by qPCR, n=4. **(b)** Representative picture of Western Blots. **(c)** SOD2 protein quantitation for **(b)**, n=5. **(d)** ROS formation, n=5. **(e)** 3-Nitrotyrosine formation, n=5. **(f)** 8-OHdG formation, n=5. **(g)** Representative pictures for γH2AX formation. **(h)** Protein quantitation for **(g)**, n=5. **(i)** Apoptosis rate by TUNEL assay, n=5. **(j)** Caspase-3 activity, n=5. **(k)** Intracellular ATP level, n=5. **(l)** ∆ᴪ m by TMRE fluorescence, n=5. **(m-o)** EBV-negative tumor cell line SZBL1 and PBMCs were treated as mentioned in **(a)** and then harvested for further analysis. **(m)** ROS formation, n=5. **(n)** 8-OHdG formation, n=5. **(o)** Apoptosis rate by TUNEL assay, n=5. *, *P*<0.05, vs CTL group; ¶, *P*<0.05, vs BA group; #, *P*<0.05, vs BA/CDM group. Results are expressed as mean ± SEM.

**Figure 5:**
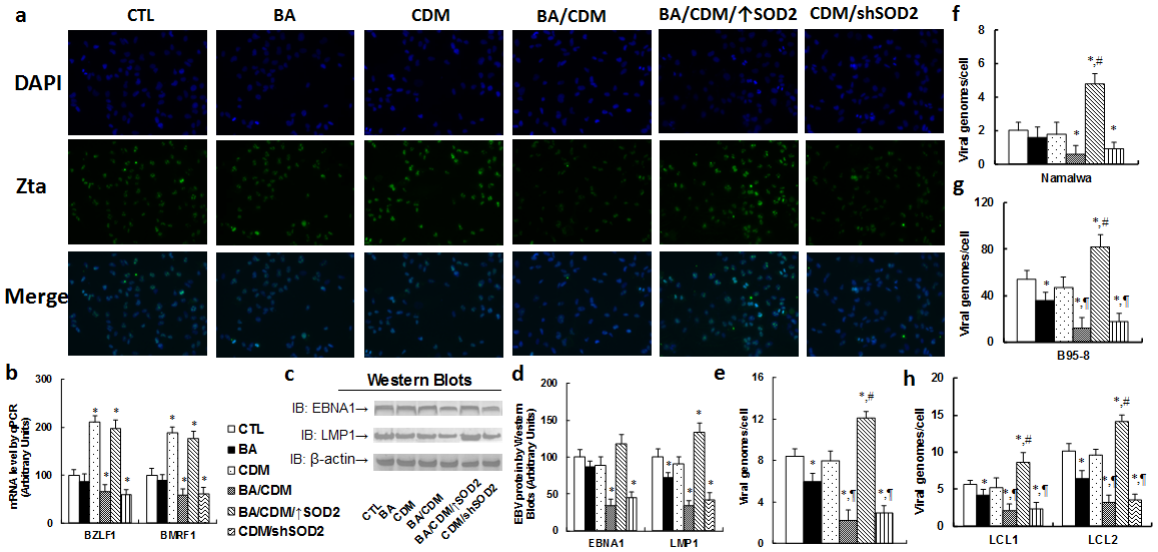
Combination of betulinic acid (BA) and chidamide (CDM) synergistically suppresses EBV activation and replication in EBV-positive tumor cells, while SOD2 overexpression diminishes this effect **(a-e)** The LCL cells were treated with control (CTL) alone, 15μg/ml BA alone (BA), 3μM CDM alone (CDM), a combination of BA and CDM (BA/CDM), a combination of BA and CDM treatment in SOD2 overexpression cells (BA/CDM/↑SOD2), or CDM treatment in SOD2 knockdown (CDM/shSOD2) cells for 24 hours, and the cells were harvested for further analysis. **(a)** Representative pictures for immunofluorescent staining showing the expression of Zta (green signal), and the cell nuclei was stained by DAPI (blue signals). **(b)** mRNA level by qPCR for BZLF1 and BMRF1, n=4. **(c)** Representative pictures of western blots for EBNA1 and LMP1 protein. **(d)** Protein quantitation for **(c)**, n=5. **(e)** EBV viral genomes/cell by qPCR, n=4. **(f-h)** Different EBV-positive tumor cells were treated as indicated in **(a)**, and the EBV genomes/cell was quantified by qPCR for Namalwa cells **(f)**, B95-8 cells **(g)**, and EBV-transformed LCL1 and LCL2 cells **(h)**, n=5. *, *P*<0.05, vs CTL group; ¶, *P*<0.05, vs BA group; #, *P*<0.05, vs BA/CDM group. Results are expressed as mean ± SEM.

The EBV-transformed LCL cells were treated with either control (CTL) alone, 15μg/ml BA alone (BA), 3μM CDM alone (CDM), a combination of BA and CDM (BA/CDM), a combination of BA and CDM treatment in SOD2 overexpression LCL cells (BA/CDM/↑SOD2), or CDM treatment in SOD2 knockdown cells (CDM/shSOD2) for 24 hours, and the cells were harvested for further analysis. We first measured the SOD2 expression, including mRNA (see Figure 4a) and protein level (see Figure 4b and 4c); the results showed that BA or BA/CDM treatment significantly suppressed SOD2 expression, CDM alone showed no effect, while BA/CDM/↑SOD2 significantly increased it, and CDM/shSOD2 significantly decreased SOD2 expression, indicating a successful lentivirus-mediated SOD2 manipulation in LCL cells. We then measured the oxidative stress, including ROS (see Figure 4d) and 3-Nitrotyrosine (see Figure 4e) formation, and DNA damage, including 8-OHdG (see Figure 4f) and γH2AX (see Figure 4g, 4h) formation. It showed that either BA or CDM alone increased oxidative stress and DNA damage, and combination of BA/CDM further potentiate the effect. Overexpression of SOD2 significantly diminished this effect, while SOD2 knockdown worsened the problem. We then measured the apoptosis rate and caspase-3 activity from the above treated LCL cells. The results showed that the apoptosis rate (see Figure 5i) and caspase-3 activity (see Figure 5j) were slightly increased with either BA or CDM treatment alone, while BA/CDM combination treatment significantly potentiate them, indicating a synergistic effect of BA and CDM. We also measured the intracellular ATP level (see Figure 5k) and mitochondrial membrane potential (Δ ᴪ m) using TMRE fluorescence (see Figure 5l), and found that both factors were decreased with either treatment of BA or CDM alone, while BA/CDM combination synergistically potentiated this effect. SOD2 overexpression partly diminished this effect, and SOD2 knockdown worsened the problem. We finally measured the effect of BA/CDM combination on the EBV-negative tumor cell line SZBL1 and primary peripheral blood mononuclear cells (PBMCs) as shown in Figure 4m-4o. It showed that BA/CDM combination significantly increased ROS formation (see Figure 4m), 8-OHdG formation (which is the marker of DNA damage; see Figure 4n), and apoptosis rate (see Figure 4o), while BA and CDM alone had a much smaller effect. Furthermore, the EBV-negative tumor cell line SZBL1 was much more sensitive to BA/CDM-mediated effect compared to primary PBMCs cells. This is consistent with the previous finding that BA and CDM have a much smaller toxic effect on primary cells compared to tumor cells [7, 11], and indicates that the combination of BA/CDM is specific to EBV-tumor cells, with a small side effect on human normal tissues in the potential clinical application.

### Combination of betulinic acid (BA) and chidamide (CDM) synergistically suppresses EBV activation and replication, while SOD2 overexpression diminishes this effect

We first measured the potential effect of BA and CDM on EBV lytic activation. The lytic protein Zta was stained by immunocytochemistry (see Figure 5a); it showed that BA alone had little effect, CDM alone significantly increased Zta expression, while combination of BA/CDM decreased Zta expression. SOD2 overexpression significantly diminished the effect of BA/CDM, while SOD2 knockdown (shSOD2) mimicked it. We then measured the mRNA expression for genes BZLF1 and BMRF1 (see Figure 5b), it showed an effect similar to that of Zta protein expression pattern. We also measured the protein expression of EBNA1 and LMP1, two key proteins for viral DNA replication and tumorigenesis in treated LCL cells (see Figure 5c, 5d). The results showed that either BA or CDM alone had little effect, while BA/CDM combination significantly decreased expression of EBNA1 and LMP1, and overexpression of SOD2 completely restored this effect, while shSOD2 mimicked it. We finally measured the EBV DNA genome copies in treated LCL cells (see Figure 5e). The results showed that BA alone slightly inhibited EBV replication, CDM alone had little effect, and BA/CDM combination synergistically decreased EBV replication by 80% compared to the control (CTL) group. Overexpression of SOD2 (BA/CDM/↑SOD2) completely diminished this effect and significantly increased EBV replication. On the other hand, SOD2 knockdown (CDM/shSOD2) mimicked the effect of BA/CDM group and significantly inhibited EBV replication. We also measured EBV suppression effect of BA/CDM on other kinds of EBV-positive tumor cells, including Namalwa cells (see Figure 5f), B95-8 cells (see Figure 5g), and EBV-transformed LCL1 and LCL2 cells (see Figure 5h), it showed an effect similar to that of LCL cells as shown in Figure 5e. This indicates that BA/CDM-mediated ROS over-generation suppresses the EBV replications.

### Combination of betulinic acid (BA) and chidamide (CDM) synergistically potentiates oxidative stress and suppresses EBV replication and tumor growth in *in vivo* xenograft tumor development, while SOD2 overexpression diminishes this effect

**Figure 6:**
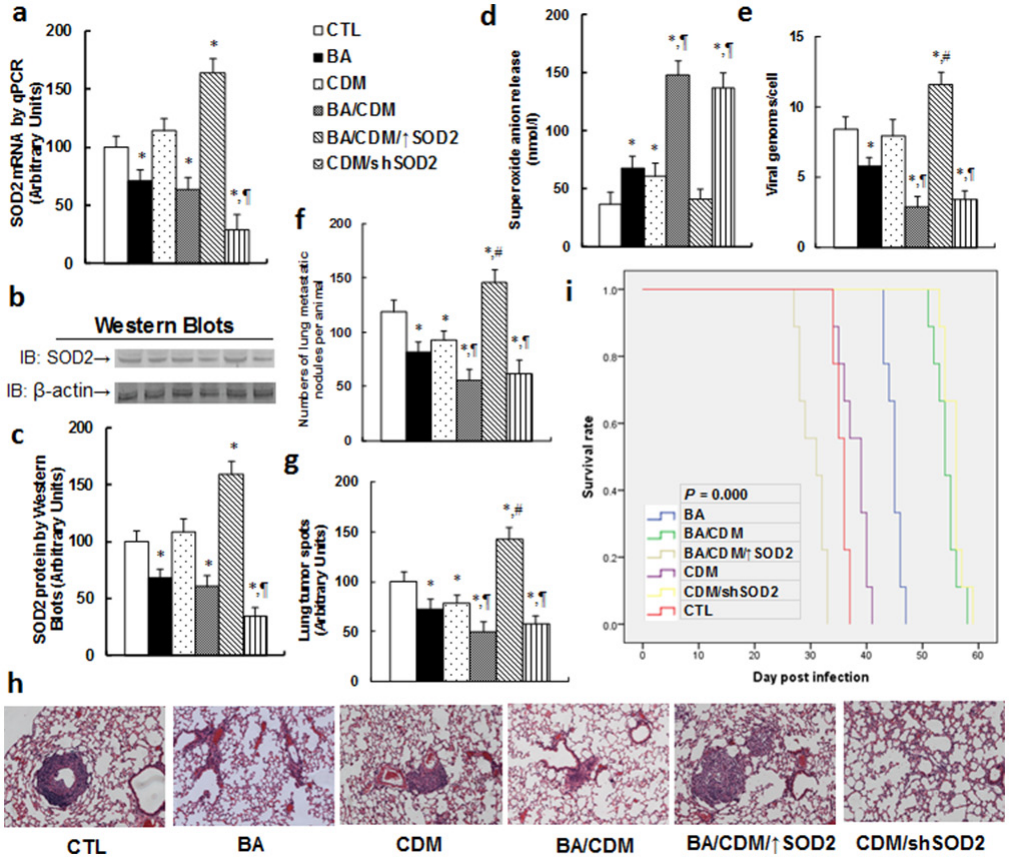
Combination of betulinic acid (BA) and chidamide (CDM) synergistically potentiates oxidative stress and suppresses EBV replication and tumor growth in *in vivo* xenograft tumor development, while SOD2 overexpression diminishes this effect The nude mice were injected with EBV-transformed LCL cells for *in vivo* xenograft tumor development study, and then treated with BA or CDM alone, a BA/CDM combination, a BA/CDM combination with SOD2 overexpressed LCL cells (BA/CDM/↑SOD2), or CDM with SOD2 knockdown LCL cells (CDM/shSOD2), and then the treated mice were sacrificed for further analysis. **(a)** SOD2 mRNA level by qPCR, n=4. **(b)** Representative pictures for SOD2 protein. **(c)** Quantitation of SOD2 protein by Western Blots, n=5. **(d)** Superoxide anion release from tumor tissues, n=5. **(e)** EBV viral genomes/cell by qPCR, n=5. **(f)** Tumor colony formation in lung, n=5. **(g)** Mice were killed upon 20% weight loss, and organs were harvested for terminal analysis. Formalin-fixed, paraffin-embedded tumor tissue from lung were sectioned to 4mm thickness, and the histopathological analyses were performed with H&E staining. Images were taken using a Carl Zeiss MIRAX MIDI slide scanner, and the lung tumor spots were analyzed using a 3DHISTECH Pannoramic Viewer, n=5. **(h)** Representative picture by H&E staining for **(g)**. **(i)** Kaplan-Meier analysis comparing survival of mice between each treatment group, *P* value represents log-rank Mantel-Cox test result, n=9. *, *P*<0.05, vs CTL group; ¶, *P*<0.05, vs BA group; #, *P*<0.05, vs BA/CDM group. Results are expressed as mean ± SEM.

We evaluated the inhibition effect of BA and CDM on EBV replication through an *in vivo* xenograft tumor development study using EBV-transformed LCL cells, and we also investigated the potential role of SOD2 through lentivirus-carried SOD2 overexpression or knockdown in LCL cells. In Figure 6, the nude mice were injected through the tail vein with LCL cells or SOD2 overexpression/knockdown LCL cells, and then were treated with either BA or CDM alone, or a BA/CDM combination. The subsequent xenograft tumor tissues from the lungs were isolated and analyzed and mouse survival was calculated. We first measured the SOD2 expression from the tumor tissues, including mRNA (see Figure 6a) and protein level (see Figure 6b, 6c). The results showed that either BA alone, or BA/CDM combination treatment, slightly decreased SOD2 expression. CDM alone showed no effect, while BA/CDM combination treatment with injection of SOD2 overexpressed LCL cells (BA/CDM/↑SOD2) showed significantly increased SOD2 expression, and CDM treatment with injection of SOD2 knockdown LCL cells (CDM/shSOD2) decreased SOD2 expression, indicating lentivirus-carried SOD2 manipulation in LCL cells and *in vivo* chemical treatment work efficiently. We then measured the superoxide anion (O_2_^-.^) release from the xenograft tumor tissues. In Figure 6d, BA or CDM alone slightly increased superoxide anion release, and the BA/CDM combination synergistically increased superoxide anion release by almost 3 times compared to the control (CTL) group. Overexpression of SOD2 (BA/CDM/↑SOD2) significantly diminished BA/CDM-mediated O_2_^-.^ release, while SOD2 knockdown (CDM/shSOD2) largely increased O_2_^-.^ release and mimicked the effect of BA/CDM combination treatment. We then measured the EBV DNA copies from those tumor tissues (see Figure 6e). We found that BA alone slightly inhibited EBV replication, CDM had no effect, and BA/CDM combination synergistically inhibited EBV replication by more than 70%, while SOD2 overexpression in the BA/CDM combination (BA/CDM/↑SOD2) significantly increased EBV replication, and SOD2 knockdown in CDM treatment (CDM/shSOD2) mimicked the effect of BA/CDM, inhibiting EBV replication by ∼35%. We then measured the lung tumor nodules formation (see Figure 6f) and lung tumor spots by H&E staining (see Figure 6g, 6h). We found that BA or CDM alone slightly decreased tumor formation, BA/CDM combination synergistically suppressed tumor formation, SOD2 expression (BA/CDM/↑SOD2) completely diminished the inhibition effect of BA/CDM and largely potentiated the tumor growth, and SOD2 knockdown (CDM/shSOD2) significantly suppressed tumor growth, mimicking the effect of BA/CDM. We finally measured the mouse survival rate using Kaplan-Meier analysis (see Figure 6i). The results showed that CDM alone slightly increased mouse survival, BA significantly increased it, and BA/CDM combination and SOD2 knockdown (CDM/shSOD2) treatment synergistically further increased mouse survival [26]. On the other hand, SOD2 overexpression (BA/CDM/↑SOD2) completely diminished the effect of BA/CDM and significantly decreased mouse survival rate compared to the control (CTL) group. The results indicate that BA/CDM combination could synergistically inhibit EBV replication and suppress tumor growth, SOD2 knockdown mimics this effect, and SOD2 overexpression diminishes this inhibition effect, worsening the problem.

## DISCUSSION

We show that BA and CDM alone have little effect on the inhibition of EBV replication, while combination of BA and CDM synergistically induces ROS generation and DNA damage and significantly inhibits EBV replication in EBV-transformed LCL cells. Overexpression of SOD2 significantly diminishes this effect, while SOD2 knockdown mimics it. The *in vivo* xenograft tumor study further proves that the combination of BA and CDM could be an efficient strategy in the removal of the EBV virus.

### Betulinic acid-mediated inhibition effect on EBV replication

It has been reported that BA could significantly inhibit the hepatitis B virus through SOD2 suppression and the subsequent ROS generation [7]. While our results showed that BA alone could only slightly inhibit EBV replication from both the *in vitro* and *in vivo* study. This could be explained because EBV is in the latent stage, and is not sensitive to the BA-mediated ROS generation and subsequent DNA damage (8, 27, 28). On the other hand, the EBV may be much more sensitive to ROS-mediated DNA damage during the replication stage [24].

### Chidamide-induced EBV activation and ROS generation

We have shown that CDM could significantly increase the expression of BZLF1 (coding Zta protein) and BMRF1 (coding for EA-D protein), which are two key proteins that are responsible for the activation of EBV from the latent to lytic stage [23, 29]. Further investigation shows that this activation is due to CDM (CS055)-mediated increased H3 acetylation on those promoters, which is consistent with previous findings that some HDACi, especially the CDM (CS055) analogue MS275, act as an inducer of EBV lytic-phase gene expression [3]. On the other hand, the *in vitro* experiments show that treatment of CDM alone only slightly increases EBV replication. In low doses of CDM, EBV replication exhibits a dose response to CDM, while in high doses of 4μM CDM, the CDM-mediated EBV replication was not increased but rather inhibited compared to 3μM CDM (see Figure 3f). This can be explained because the CDM-mediated EBV replication was inhibited by higher doses of CDM-mediated ROS generation and DNA damage [11, 25]. It has been reported that accumulation of ROS occurs in transformed cells cultured with HDACi, such as MS275 [5, 9] and CDM (CS055) [11]. In tumor cells, ROS-mediated oxidation-reduction pathways are important mechanisms for HDACi-induced cell death [30, 31].

### Combination of BA/CDM-mediated inhibition effect on EBV replication

We have shown that BA alone has only a slight inhibition effect on EBV replication through generation of ROS and subsequent DAN damage, while CDM alone (*in vitro* treatment with low dose) does not inhibit but rather slightly increases EBV replication, even though CDM has been widely reported to be able to inhibit tumor growth [10, 11]. Interestingly, the *in vivo* experiments show that CDM alone has little inhibition effect on both EBV replication and EBV-transformed LCL tumor growth (see Figure 6), which may be explained because CDM treatment-induced apoptosis and tumor inhibition was partly normalized by CDM-mediated EBV replication. On the other hand, both *in vitro* and *in vivo* experiments show that BA/CDM combination significantly suppressed EBV replication and EBV-transformed LCL tumor growth. This can be explained because CDM-mediated EBV activation and gene expression sensitizes the EBV to be the target of ROS over-generation that is induced by the combination of BA/CDM. ROS overgeneration-mediated DNA damage eventually inhibits EBV replication and ROS overgeneration-mediated apoptosis and cell death suppresses the EBV-transformed LCL tumor growth.

In addition, our results showed that BA/CDM-mediated ROS over-generation suppressed EBV-genome replication in LCL cells, and overexpression of SOD2 completely diminished this effect, indicating that ROS plays a major role in BA/CDM-mediated suppression of EBV replication. On the other hand, some literatures have also reported that ROS formation induces EBV reactivation [32, 33]. This discrepancy may be due to different amounts of ROS that are generated in different situations. If certain amounts (not too much) of ROS are generated, ROS will activate Nrf2 (NF-E2-related factor 2), a major oxidative stress regulator [34], and subsequently upregulates the expression of downstream antioxidant genes, such as SOD2, resulting in EBV activation. On the other hand, if too much ROS is generated, it will suppress rather than activate Nrf2, and the suppressed Nrf2 will further suppress the downstream antioxidant gene expression, leading to a negative forward loop with even worse oxidative stress that eventually results in apoptosis and the suppression of EBV replication.

## CONCLUSIONS

**Figure 7:**
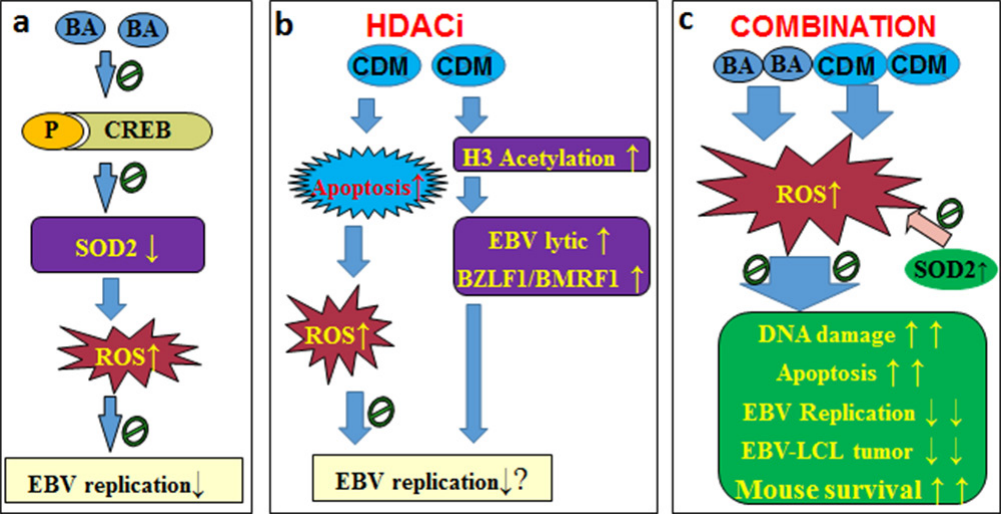
Proposed mechanisms for BA/CDM combination-mediated synergistic inhibition effect on EBV replication (a) BA alone slightly suppresses EBV replication. **(b)** CDM alone has little effect on EBV replication. **(c)** BA/CDM combination synergistically increases ROS generation, significantly inhibits EBV replication and LCL-tumor growth with prolonged mouse survival, overexpression of SOD2 diminishes this effect. Abbreviations: BA: betulinic acid; CDM: chidamide (CS055); CREB: cAMP-response element binding protein; EBV: Epstein-barr virus; H3: histone 3; HDACi: histone deacetylase inhibitors; LCL: lymphoblastoid cell line; P: phosphorylation; ROS: reactive oxygen species; SOD2: mitochondrial superoxide dismutase.

Taken together, the mechanism for BA/CDM combination-mediated synergistic inhibition effect on EBV replication can be conceptualized in Figure 7. In Figure 7a, the BA alone suppresses SOD2 expression with increased ROS formation, and slightly inhibits EBV replication. In Figure 7b, the histone deacetylase inhibitor CDM increases H3 acetylation and activates the EBV lytic gene (BZLF1 and BMRF1) expression, while CDM-mediated ROS formation and apoptosis suppresses the CDM-mediated EBV activation. In Figure 7c, the BA/CDM combination synergistically increases ROS generation with DNA damage and LCL apoptosis, and synergistically inhibits EBV replication and LCL tumor growth with prolonged mouse survival, while SOD2 overexpression diminishes this effect. We conclude that a combination of betulinic acid and chidamide synergistically inhibits Epstein-Barr virus replication through over-generation of reactive oxygen species. This may be an efficient strategy for the clinical removal of EBV infection.

## EXPERIMENTAL PROCEDURES MATERIALS AND METHODS

Antibodies for β-actin (sc-47778) and SOD2 (sc-30080) were obtained from Santa Cruz Biotechnology. Antibodies for acetyl-histone H3 Lys14 (#07-353) and Lys18 (#04-1107), acetyl-histone H3 (#06-599) and histone H3 (#05-499) were obtained from EMD Millipore. H2AX (ab20669) and γH2AX (ab2893) were obtained from Abcam, 3-nitrotyrosine (3-NT) was measured by 3-Nitrotyrosine ELISA Kit (ab116691 from Abcam) per manufacturers’ instructions. The siRNA for CREB-1 (# s3491) and negative control (# AM4636) were obtained from Ambion.

The mitochondrial fraction was isolated using a Pierce Mitochondria Isolation Kit (Pierce Biotechnology) per manufacturers’ instructions. Nuclear extracts were prepared using the NE-PER Nuclear and Cytoplasmic Extraction Reagents Kit (Pierce Biotechnology). Protein concentration was measured using the Coomassie Protein Assay Kit (Pierce Biotechnology). The plasmid DNA and siRNA were transfected by Lipofectamine™ Reagent (Invitrogen). Luciferase activity assay was carried out using the Dual-Luciferase™ Assay System (Promega) and the transfection efficiency was normalized using a cotransfected renilla plasmid.

Chidamide (CDM, CS055) was supplied by Chipscreen Biosciences (Shenzhen, China) and was dissolved in DMF (dimethyl-formamide). For the *in vivo* experiments, chidamide was suspended in 0.1% sodium carboxyl methylcellulose and stored at 4°C. Betulinic acid (BA) was purchased from Sigma, and the compounds were dissolved in DMSO (dimethyl sulfoxide) to make a stock solution. The final concentration of the above solvents did not exceed 0.5% in any experiment.

### Cell culture and chemical treatment

The B95-8 and Namalwa cell lines were kind gifts of Dr Haimou Zhang (Hubei University). The B95-8 cell line was used for studies of immortalization and viral production. Our lab has generated 3 EBV-transformed lymphoblastoid cell lines, which were named LCL, LCL1, LCL2, respectively [2, 35]. Namalwa cell line is a BL-cell line that contains two copies of the EBV genome, and was used to generate the standard curve for calculation of EBV genome copy number [36, 37]. SZBL1 was EBV-negative B-cell lymphoma cell line prepared in our lab as virus-negative controls. The Peripheral Blood Mononuclear Cells (PBMCc) were used as non-EBV and primary healthy cells for the negative control. All the cells were maintained in RPMI 1640 culture medium with 10% fetal bovine serum (FBS) at 37°C with 5% CO_2_. To induce the EBV lytic cycle, different doses of betulinic acid (BA) and/or chidamide (CDM) were added in culture medium for 24 hours and then harvested for further analysis. This duration of stimulus and all doses of chemicals were determined to be in the optimal range for a maximal response.

### Construction of plasmids and vectors

The EBV genomic DNA was prepared from the above LCLs. In order to construct BZLF1 reporter plasmid, the BZLF1 gene promoter (nt101690- nt102626 from GenBank: V01555.2) was amplified by PCR and subcloned into pGL3-basic vector (# E1751, Promega) using restriction sites of KpnI and Xho1 with the following primers: Forward: 5’-gcgc-ggtacc- cat aaa aat aag tgc atg gat -3’ (Kpn I) and Reverse: 5’- tcga-ctcgag - gcg gac ggt ggg gct cat gga -3’ (Xho1). In order to construct BMRF1 reporter plasmid, the BMRF1 gene promoter (nt77899- nt80399 from GenBank: V01555.2) was amplified by PCR and subcloned into pGL3-basic vector using restriction sites of Xho1 and HindIII with the following primers: Forward: 5’- tcga-ctcgag - atg tct ttg cgg acc tgg gct -3’ (Xho1) and Reverse: 5’- gtac- aagctt- caa cag cac ctg ccc aat cat -3’ (Hind III). In order to construct the CREB expression plasmid, the human cDNA for CREB (from Open Biosystems) was amplified by PCR and subcloned into pcDNA3.1 using restriction sites of HindIII and BamH1 with the following primers: Forward: 5’- gtac- aagctt- atg acc atg gaa tct gga gcc -3’ (Hind III) and Reverse: 5’-gcgc- ggatcc – tta atc tga ttt gtg gca gta -3’ (BamH I). All the vectors were verified by sequencing, and detailed information on thee plasmids is available upon request.

### Measurement of ROS generation

Treated cells were seeded in a 96-well plate and incubated with 10μM CM-H2DCFDA (Invitrogen) for 45 min at 37°C, and then the intracellular formation of reactive oxygen species (ROS) was measured at excitation/emission wavelengths of 485/530nm using a FLx800 microplate fluorescence reader (Bio-Tek). The data was normalized as arbitrary units [38]. Levels of oxidative marker 3-nitrotyrosine (3-NT) were measured by western blots.

### Measurement of DNA breaks

8-OHdG formation was measured using an OxiSelect™ Oxidative DNA Damage ELISA Kit (Cat No. STA320, from Cell Biolabs Inc.) per manufacturers’ instructions. The formation of γH2AX was measured from nuclear extracts by western blotting using H2AX as input control.

### Evaluation of SOD2 activity

The SOD activity from the mitochondrial fraction of LCL cells was measured as described previously [39]. In brief, a stable O_2_^.-^ source was generated through the conversion action of XOD (xanthine oxidase) from xanthine and was mixed with chemiluminescent (CL) reagents to achieve a stable light emission. The SOD2 sample injection can scavenge O_2_^.-^ and the subsequent decrease of chemiluminescent response is proportional to the SOD2 activity. This system can have a detection limit of 0.001U.ml^-1^ with the linear range of 0.03∼2.00U.ml^-1^. The results were normalized by protein concentration and were expressed as Units/mg proteins (U/mg).

### RT reaction and real-time quantitative PCR

Total RNA from treated cells was extracted using the RNeasy Micro Kit (Qiagen), and the RNA was reverse transcribed using an Omniscript RT kit (Qiagen). All the primers were designed using Primer 3 Plus software with the Tm at 60°C, primer size as 21bp, and the product length in the range of 140-160bp (see Table 1). The primers were validated with the amplification efficiency in the range of 1.9-2.1, and the amplified products were confirmed with agarose gel. The real-time quantitative PCR was run on iCycler iQ (Bio-Rad) with the Quantitect SYBR green PCR kit (Qiagen). The PCR was performed by denaturing at 95°C for 8 min, followed by 45 cycles of denaturation at 95°C, annealing at 60°C, and extension at 72°C for 10s, respectively. 1 μl of each cDNA was used to measure target genes. The β-actin was used as the housekeeping gene for transcript normalization, and the mean values were used to calculate relative transcript levels with the ^ΔΔ^CT method per instructions from Qiagen. In brief, the amplified transcripts were quantified by the comparative threshold cycle method using β-actin as a normalizer. Fold changes in gene mRNA expression were calculated as 2^−ΔΔCT^ with CT = threshold cycle, ΔCT=CT(target gene)-CT(β-actin), and the ΔΔCT =ΔCT(experimental)-ΔCT (reference).

**Table 1:** Sequences of primers for the real time quantitative PCR (qPCR)

Gene	Species	Analysis	Forward primer (5'→3')	Reverse primer (5'→3')
β-actin	Human	mRNA	gatgcagaaggagatcactgc	atactcctgcttgctgatcca
SOD2	Human	mRNA	gcctacgtgaacaacctgaac	tgaggtttgtccagaaaatgc
BZLF1	EBV	mRNA	gggggataatggagtcaacat	tagcgtcccaaacataaatgc
BMRF1	EBV	mRNA	tcctgtccaagtgctatgacc	gggagacctcgaagctgatta
BZLF1	EBV	ChIP	ttttggggtacctgaaatgtg	gggtacccaaaccaaatgtaga
BMRF1	EBV	ChIP	gcgagccataaagcagtttct	atcggaactctcttgctcaaa
β-actin	Human	Genome	ctggacttcgagcaagagatg	aggaaggaaggctggaagagt
BMRF1	EBV	Genome	ccgtcctgtccaagtgctat	gggagacctcgaagctgatta

### Western blotting

Cells were lysed in an ice-cold lysis buffer (0.137M NaCl, 2mM EDTA, 10% glycerol, 1% NP-40, 20mM Tris base, pH 8.0) with protease inhibitor cocktail (Sigma). The proteins were separated in 10% SDS-PAGE and further transferred to the PVDF membrane. The membrane was incubated with appropriate antibodies, washed and incubated with HRP-labeled secondary antibodies, and then the blots were visualized using the ECL+plus Western Blotting Detection System (Amersham). The blots were quantitated by IMAGEQUANT, and the results were normalized by β-actin.

### Luciferase reporter assay

1.0×10^5^ cells were seeded in a 6-well plate with completed medium to grow until they reached 80% confluence. The related luciferase reporter plasmids (3μg) and 0.2μg pRL-CMV-Luc *Renilla* plasmid (from Promega) were transiently cotransfected, and in some experiments, the siRNA oligoneucleotides were cotransfected. After treatment, the cells were harvested and the luciferase activity assays were carried out using the Dual-Luciferase^TM^ Assay System (Promega), and the transfection efficiencies were normalized using a cotransfected *Renilla* plasmid per manufacturers’ instructions.

### Chromatin immunoprecipitation (ChIP)

Cells were washed and crosslinked using 1% formaldehyde for 20 min and terminated by 0.1M glycine. Cell lysates were sonicated and centrifuged. 500μg of protein were pre-cleared by BSA/salmon sperm DNA with preimmune IgG and a slurry of protein A agarose beads. Immunoprecipitations were performed with the indicated antibodies, BSA/salmon sperm DNA and a 50% slurry of protein A agarose beads. Input and immunoprecipitates were washed and eluted, and then incubated with 0.2mg/ml Proteinase K for 2h at 42°C, followed by 6h at 65°C to reverse the formaldehyde crosslinking. DNA fragments were recovered by phenol/chloroform extraction and ethanol precipitation. A 140-160bp fragment in the range of -300∼-100 from the transcription start site on BZLF1 or BMRF1 promoter was amplified by real-time PCR (qPCR) using the primers indicated in Table 1.

### Measurement of apoptosis

Apoptosis was evaluated by TUNEL assay using the *In Situ* Cell Death Detection Kit™ (Roche). Cells were fixed in 4% paraformaldehyde and labeled by TUNEL reagents. Stained cells were photographed by a fluorescence microscope and further quantified by FACS analysis. Caspase-3 activity was determined by the ApoAlert caspase assay kit (Clontech). Treated cells were harvested and 50 μg of proteins were incubated with the fluorogenic peptide substrate Ac-DEVD-7-amino-4-trifluoromethyl coumarin (AFC). The initial rate of free AFC release was measured using a FLx800 microplate reader (Bio-Tek) at excitation/emission wavelengths of 380/505nm, and enzyme activity was calculated as pmol/min/mg [38].

### Measurement of mitochondrial function

The intracellular ATP level was determined by the luciferin/luciferase-induced bioluminescence system. An ATP standard curve was generated at concentrations of 10^-12^-10^-3^M. Intracellular ATP levels were calculated and expressed as nmol/mg protein. The mitochondrial membrane potential (Δψm) was measured by TMRE (from Molecular Probes T-669) staining. A 600μM T-669 stock solution was prepared using DMSO. Cells were grown on coverslips and immersed in 600nM TMRE for 20 min at 37°C to load them with dye. The labeling medium was then aspirated and the cells were immersed in 150nM TMRE to maintain the equilibrium distribution of the fluorophore. The coverslips were mounted with live cells onto confocal microscopes to image the cells using 548nm excitation/573nm emission filters. The intensity of TMRE fluorescence was measured using Image J software. Data from 10-20 cells were collected for each experimental condition and mean values of fluorescence intensity ± SEM were calculated [40].

### Detection of EBV copy number

The genomic DNA was extracted from either *in vitro* EBV-tumor cells or EBV-transformed LCL tumor tissue in mice using a QIAamp DNA Mini Kit (Qiagen). The EBV DNA copy number was measured by qPCR using 50ng of total DNA with EBV BMRF1 primers (see Table 1), and the results were normalized by cellular β-actin (primers see Table 1) as an internal control [18, 41]. The Namalwa cell line, which contains 2 EBV viral genome copies, was used as a standard to prepare calibration curves for both EBV BMRF1 and β-actin genes. The EBV viral load was presented as the number of viral genomes per cell [36, 37].

### Immunocytochemistry

EBV-positive LCL cells grown on cover slips coated with 0.1% gelatin were treated with either control (CTL) alone, 15μg/ml BA alone (BA), 3μM CDM alone (CDM), or a combination of BA and CDM (BA/CDM) for 24 hours. Cells were fixed with acetone for 10 min at room temperature. The fixed cells were then stained with anti-Zta (1:50) mouse monoclonal antibodies overnight at 4°C, and then further stained by Goat Anti-Mouse IgG H&L (Alexa Fluor® 488, obtained from Abcam #ab150113). The expression of EBV lytic proteins was visualized under fluorescence microscopy, and the nuclei of cells were stained with 40, 6-diamidino-2-phenylindole (DAPI) [36, 42].

### Generation of SOD2 lentivirus expression LCLs

The human SOD2 cDNA (obtained from Open Biosystems) was amplified by PCR, and then subcloned into the pLVX-Puro vector (from Clontech) using restriction sites of Xho1 and Xba1 with the following primers: Forward: 5’- tcga-ctcgag- atg ttg agc cgg gca gtg tgc -3’ (Xho I) and Reverse: 5’- gcgc-tctaga- tta ctt ttt gca agc cat gta -3’ (Xba I), and the Empty (CTL) or SOD2 lentivirus was expressed through Lenti-X™ Lentiviral Expression Systems (from Clontech) per manufacturers’ instructions. The virus for SOD2 expression and related empty (EMP) were used to infect LCLs, the positive clones were selected by 10μg/ml puromycin, the single colony was picked up, and the SOD2 expression efficiency was confirmed by real time PCR and western blotting. The stable SOD2 expression LCL cells were used for *in vivo* mice xenograft tumor study.

### Generation of SOD2 lentivirus knockout LCLs

According to our preliminary data from *in vitro* cell culture experiments, the following sequence was confirmed as the most effective to knockdown human SOD2: Top strand: 5’- CACC gcc tgc act gaa gtt caa tgg CGAA cca ttg aac ttc agt gca ggc -3’, and the bottom strand: AAAA gcc tgc act gaa gtt caa tgg TTCG cca ttg aac ttc agt gca ggc -3’. The shRNA template for SOD2 or scrambled were designed (sense strand + loop + antisense strand) and the related double strand DNA (dsDNA) was synthesized and annealed. They were subcloned into the pLVX-shRNA1 vector (from Clontech) using BamH1/EcoR1 restriction sites. The lentivirus was expressed through Lenti-X™ shRNA Expression Systems (from Clontech) per manufacturers’ instructions. The virus for shSOD2 and related empty (EMP) was used to infect LCLs, the positive clones were selected by 10μg/ml puromycin, the single colony was picked up, and the knockout efficiency was confirmed by real time PCR and western blotting. The stable SOD2 knockout LCL (shSOD2) cells were used for *in vivo* mice xenograft tumor study.

### *In vivo* xenograft EBV-tumor study

The Balb/c athymic nude male mice (6 weeks old) were obtained from the Disease Prevention Center of Guangdong Province. All procedures involving mice were conducted in accordance with NIH regulations concerning the use and care of experimental animals, and were approved by the Institutional Animal Care and Use Committee (from Guangdong Medical University and Wuhan University). The 2x10^6^ viable EBV-transformed lentivirus infected LCL cells were washed, harvested in PBS, and then injected into the lateral tail vein in a volume of 0.1ml. After 2 days of the implantation of the primary xenograft, the mice were treated by 25mg/kg of body mass of either BA (corn oil as vehicle) or CDM (0.1% sodium carboxyl methylcellulose as vehicle), or a combination of BA/CDM via oral gavage 3 times a week. The mice with tail vein injection of lentivirus-infected LCL cell were separated into 6 groups (n=9). Group 1 (CTL): LCL cells (Empty lentivirus) plus treatment of chemical vehicle (corn oil + 0.1% sodium carboxyl methylcellulose); Group 2 (BA): LCL cells (Empty lentivirus) plus treatment of BA; Group 3 (CDM): LCL cells (Empty lentivirus) plus treatment of CDM; Group 4 (BA/CDM): LCL cells (Empty lentivirus) plus treatment of BA and CDM; Group 5 (BA/CDM/↑SOD2): LCL cell (SOD2 lentivirus) plus treatment of BA and CDM; Group 6 (CDM/shSOD2): LCL cell (shSOD2 lentivirus) plus treatment of CDM. Mice were monitored for changes in body weight and killed when values fell below 20% of their initial weight. The lungs from sacrificed mice were isolated and fixed in 10% formalin. The number of surface metastases per lung was determined under a dissecting microscope. Formalin-fixed, paraffin-embedded tumor tissue from the lungs were sectioned to 4 mm thickness, and the histopathological analyses were performed with H&E staining. Images were taken using a Carl Zeiss MIRAX MIDI slide scanner, and analyses were performed using a 3DHISTECH Pannoramic Viewer. The tumor tissues were isolated for *in vivo* monitoring of superoxide anion release, and the SOD2 expression from tumor tissues were measured by real time PCR for mRNA and Western Blotting for protein level.

### *In vivo* superoxide release

The superoxide anion (O_2_^.-^) release from the tumor tissue was determined by a luminol-EDTA-Fe enhanced chemiluminescence (CL) systemsupplemented with DMSO-TBAC (Dimethyl sulfoxide-tetrabutyl-ammonium chloride) solution for extraction of released O_2_^.-^ from tissues as described previously [38]. The superoxide levels were calculated from the standard curve generated by the xanthine/xanthine oxidase reaction.

### Statistical analysis

The data was given as mean ± SEM, and all of the experiments were performed at least in quadruplicate unless otherwise indicated. The one-way or two-way ANOVA followed by the Bonferroni post hoc test was used to determine statistical significance of different groups, and the mouse survival curve was determined by Kaplan-Meier survival analysis using SPSS 22 software, and a *P* value < 0.05 was considered significant.
